# A single-nucleus transcriptomic atlas of the dog hippocampus reveals the potential relationship between specific cell types and domestication

**DOI:** 10.1093/nsr/nwac147

**Published:** 2022-07-23

**Authors:** Qi-Jun Zhou, Xingyan Liu, Longlong Zhang, Rong Wang, Tingting Yin, Xiaolu Li, Guimei Li, Yuqi He, Zhaoli Ding, Pengcheng Ma, Shi-Zhi Wang, Bingyu Mao, Shihua Zhang, Guo-Dong Wang

**Affiliations:** State Key Laboratory of Genetic Resources and Evolution, Kunming Institute of Zoology, Chinese Academy of Sciences, Kunming 650223, China; NCMIS, CEMS, RCSDS, Academy of Mathematics and Systems Science, Chinese Academy of Sciences, Beijing 100190, China; School of Mathematical Sciences, University of Chinese Academy of Sciences, Beijing 100049, China; State Key Laboratory of Genetic Resources and Evolution, Kunming Institute of Zoology, Chinese Academy of Sciences, Kunming 650223, China; Kunming College of Life Science, University of Chinese Academy of Sciences, Kunming 650223, China; State Key Laboratory of Genetic Resources and Evolution, Kunming Institute of Zoology, Chinese Academy of Sciences, Kunming 650223, China; College of Life Science, University of Chinese Academy of Sciences, Beijing 100049, China; Kunming College of Life Science, University of Chinese Academy of Sciences, Kunming 650223, China; State Key Laboratory of Genetic Resources and Evolution, Kunming Institute of Zoology, Chinese Academy of Sciences, Kunming 650223, China; Genomic Center of Biodiversity, Kunming Institute of Zoology, Chinese Academy of Sciences, Kunming 650223, China; State Key Laboratory of Genetic Resources and Evolution, Kunming Institute of Zoology, Chinese Academy of Sciences, Kunming 650223, China; Genomic Center of Biodiversity, Kunming Institute of Zoology, Chinese Academy of Sciences, Kunming 650223, China; Genomic Center of Biodiversity, Kunming Institute of Zoology, Chinese Academy of Sciences, Kunming 650223, China; State Key Laboratory of Genetic Resources and Evolution, Kunming Institute of Zoology, Chinese Academy of Sciences, Kunming 650223, China; State Key Laboratory of Genetic Resources and Evolution, Kunming Institute of Zoology, Chinese Academy of Sciences, Kunming 650223, China; State Key Laboratory of Genetic Resources and Evolution, Kunming Institute of Zoology, Chinese Academy of Sciences, Kunming 650223, China; Center for Excellence in Animal Evolution and Genetics, Chinese Academy of Sciences, Kunming 650223, China; NCMIS, CEMS, RCSDS, Academy of Mathematics and Systems Science, Chinese Academy of Sciences, Beijing 100190, China; School of Mathematical Sciences, University of Chinese Academy of Sciences, Beijing 100049, China; Center for Excellence in Animal Evolution and Genetics, Chinese Academy of Sciences, Kunming 650223, China; Key Laboratory of Systems Health Science of Zhejiang Province, School of Life Science, Hangzhou Institute for Advanced Study, University of Chinese Academy of Sciences, Hangzhou 310024, China; State Key Laboratory of Genetic Resources and Evolution, Kunming Institute of Zoology, Chinese Academy of Sciences, Kunming 650223, China; College of Life Science, University of Chinese Academy of Sciences, Beijing 100049, China; Kunming College of Life Science, University of Chinese Academy of Sciences, Kunming 650223, China; Center for Excellence in Animal Evolution and Genetics, Chinese Academy of Sciences, Kunming 650223, China

**Keywords:** dog, single-nucleus RNA sequencing, hippocampus, domestication

## Abstract

The process of domestication has led to dramatic differences in behavioral traits between domestic dogs and gray wolves. Whole-genome research found that a class of putative positively selected genes were related to various aspects of learning and memory, such as long-term potentiation and long-term depression. In this study, we constructed a single-nucleus transcriptomic atlas of the dog hippocampus to illustrate its cell types, cell lineage and molecular features. Using the transcriptomes of 105 057 nuclei from the hippocampus of a Beagle dog, we identified 26 cell clusters and a putative trajectory of oligodendrocyte development. Comparative analysis revealed a significant convergence between dog differentially expressed genes (DEGs) and putative positively selected genes (PSGs). Forty putative PSGs were DEGs in glutamatergic neurons, especially in Cluster 14, which is related to the regulation of nervous system development. In summary, this study provides a blueprint to understand the cellular mechanism of dog domestication.

## INTRODUCTION

The domestication process has led to dramatic differences in behavioral and morphological traits between domesticated animals and their wild ancestors [1–3]. Domestic dogs have undergone parallel evolution and convergent evolution with humans in the history of early hunting–gathering and the recent change of living environments from agrarian societies to modern cities [4–8]; in this process, dogs have undergone strong artificial selection, resulting in ∼450 globally recognized breeds, which makes them the most variable mammalian species on Earth [9–11]. Genome-wide scans for positive selection revealed that the behavioral and neurological traits likely changed subsequently with the process of domestication [12]. For instance, putative positively selected genes (PSGs) in dogs were reported to be linked to neural crest and central nervous system (CNS) development [13]. Gene expression in brains showed a strong difference in putative PSGs in neurons on learning, memory and behavior between domestic animals and their wild relatives [14,15].

The hippocampus is an important part of the limbic system in the brain that has an essential role in memory [16]. A class of domesticated genes is related to hippocampal synaptic long-term potentiation and long-term depression [5]. Single-cell RNA sequencing (scRNA-seq) has been used to identify the cell-type atlas [17–19], reveal cellular and molecular dynamics in neurogenesis [20] and decode hippocampal development and evolution [21]. It is worth noting that dozens of putative PSGs in dogs were related to cell types of both excitatory and inhibitory neurons and contributed to the connectivity and development of synapses and dendrites [13,15].

In the present study, we collected single nuclei from the hippocampus of a Beagle dog and performed a comprehensive analysis of the transcriptomic profiles of 105 057 nuclei. We defined 26 cell clusters and identified a set of marker genes for each cell type. Cross-species comparative analysis showed that the major cell types in the hippocampus were highly conserved between dogs and humans. More interestingly, 86 of the differentially expressed genes (DEGs) were putative PSGs during dog domestication. The comprehensive cell landscape of the dog hippocampus could help us establish correspondence between cell types in the nervous system and putative PSGs in dogs and facilitate the understanding of the molecular features of cells during domestication.

## RESULTS

### Single-nucleus transcriptomics of the dog hippocampus constructed using SPLiT-seq

**Figure 1.**
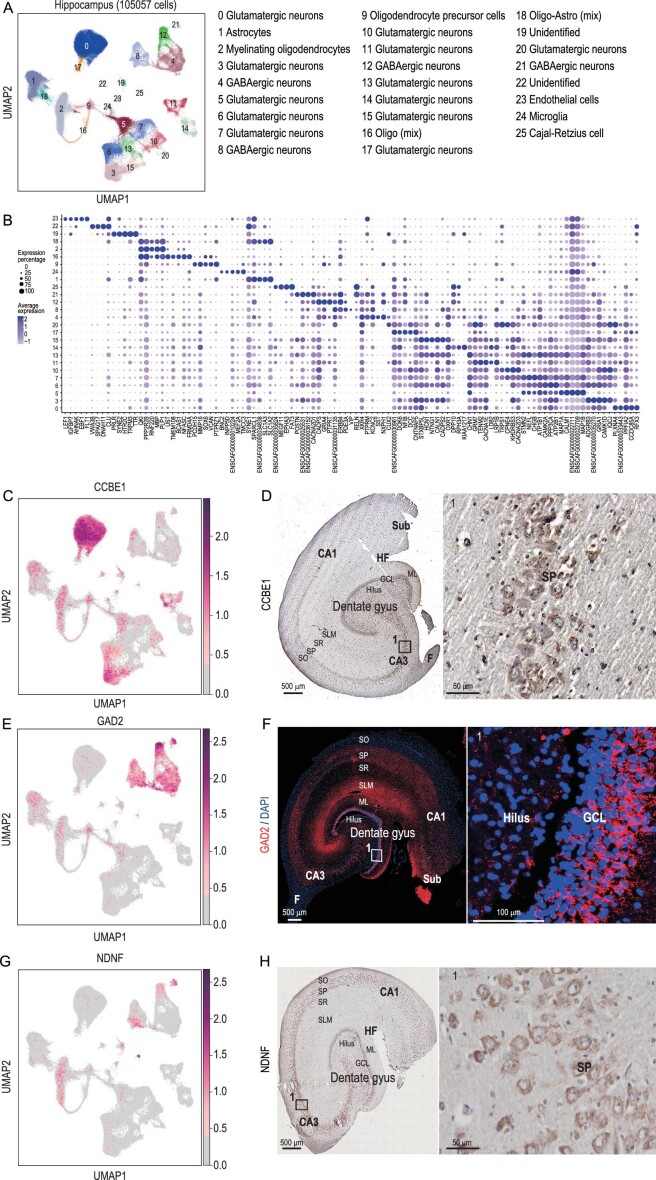
Single-nucleus transcriptomic landscape of the dog hippocampus. (A) Clustering and visualization of transcriptomes of 105 057 single cells by UMAP. In this article, ‘cluster’ means a group of cells and is related to the cluster analysis result. ‘Cell type’ is a group of clusters and we defined these clusters as the same cell types. (B) Dot plot showing the averaged DEG expression (z-cores) in different clusters (Supplementary Table S12). (C) Visualization of *CCBE1* by UMAP. (D) *CCBE1* expressed in the dog hippocampus revealed by immunohistochemical staining. (E) Visualization of *GAD2* by UMAP. (F) *GAD2* expression in the dog hippocampus revealed by immunofluorescent staining. Red: *GAD2*; blue: DAPI; white square indicates region boxed had higher-magnification view. (G) Visualization of *NDNF* by UMAP. (H) *NDNF* expression in the dog hippocampus revealed by immunohistochemical staining. Red points indicate cells that were found expressed DEGs. DG, dentate gyrus; GCL, granule cell layer, SGZ, subgranular zone; ML, molecular layer; CA, cornu ammonis; SO, stratum oriens; SP, stratum pyramidale; SR, stratum radiatum; SLM, stratum lacunosum-moleculare; Sub, subiculum; HF, hippocampus fissure; A, alveus.

A standardized single-nucleus RNA-sequencing (snRNA-seq) pipeline was built using SPLiT-seq [22]. The snRNA-seq data of single nuclei from the hippocampus of a 5-month-old Beagle dog were obtained with random and oligo(dT) primers. After detailed preprocessing and filtering (see ‘Methods’ section), we created a digital expression matrix of 105 057 single nuclei with a median of 804 genes and 1109 counts per nucleus. To remove the potential batch effects, partial principal component analysis (partial-PCA, see ‘Methods’ section) was performed instead of the classical PCA and then the uniform manifold approximation and projection method (UMAP) was applied to project these two batches of transcripts into the common comparable 2D space (Supplementary Fig. S1A). Furthermore, the Leiden community detection algorithm was employed to group these cells into 26 cell clusters (Fig. 1A).

The DEGs (*P*-value < 0.001 and log_2_(fold change) > 0.25) of each cell cluster were used to assign most of the cells to specific cell types based on the known markers in mice and humans [17,22–25] (Supplementary Table S1 and Supplementary Fig. S1B). Glutamatergic neurons, GABAergic neurons, Cajal-Retzius cells, oligodendrocyte precursor cells (OPCs), myelinating oligodendrocytes, endothelial cells, astrocytes and microglia cells were detected in the hippocampus of the Beagle at proportions of 55.82%, 12.80%, 0.14%, 3.14%, 11.68%, 0.30%, 11.82% and 0.30%, respectively (Fig. 1B and Supplementary Fig. S1B–D).

Among the clusters, 12 clusters (0, 3, 5, 6, 7, 10, 11, 13, 14, 15, 17 and 20) were identified as the glutamatergic neurons, which produce the most common excitatory neurotransmitter in the CNS [26]. Although Clusters 3, 5 and 6 had a similar expression pattern, there were unique DEGs expressed in each cluster. For instance, both Clusters 3 and 6 had high expression of *GRM7, TENM2* and *CACNA1E*, while Cluster 3 had no expression of *STMN2* or *NEFL* (Fig. 1B). *CCBE1* is a specific marker gene specifically in Cluster 0 (Fig. 1C). It marked the canine hippocampus cornu ammonis 3 (CA3) and stratum pyramidale (SP) (Fig. 1D) as the same in the mouse [27]. The GO enrichment analysis showed that Clusters 7 and 13 were enriched in the GO term cognition (GO : 0050890, Supplementary Table S2), Clusters 11 and 20 were enriched in behavior (GO : 0007610, Supplementary Table S2) and Cluster 13 was enriched in adult locomotory behavior (GO : 0008344, Supplementary Table S2).

Four clusters (4, 8, 12 and 21) are GABAergic neurons related to a kind of inhibitory neurotransmitter in the CNS, indicated by the common marker gene *GAD2* [23] (Fig. 1E). Immunofluorescence (IF) analysis showed that *GAD2* was highly expressed in the granule cell layer (GCL) and molecular layer (ML) of the dentate gyrus (DG) area and in the stratum lacunosum-moleculare (SLM) and SP of the CA (cornu ammonis) area (Fig. 1F). The GO enrichment analysis illustrated that four GABAergic neuron clusters were enriched in behavior (GO : 0007610, Supplementary Table S3). Cluster 25 was identified as the Cajal-Retzius cell, which plays a major role in cortical development [28,29]. The marker gene of the cell type was *NDNF* (Fig. 1G and H), which was also expressed in Cajal-Retzius cells in humans and mice [22,30]. Beyond the neuron cell clusters described above, another five clusters represented non-neuronal cells. Clusters 19 and 22 were unidentified types. Clusters 1, 23 and 24 were identified as astrocytes, endothelial cells and microglia cells, respectively.

### Cross-species comparison between dog and human hippocampus

**Figure 2.**
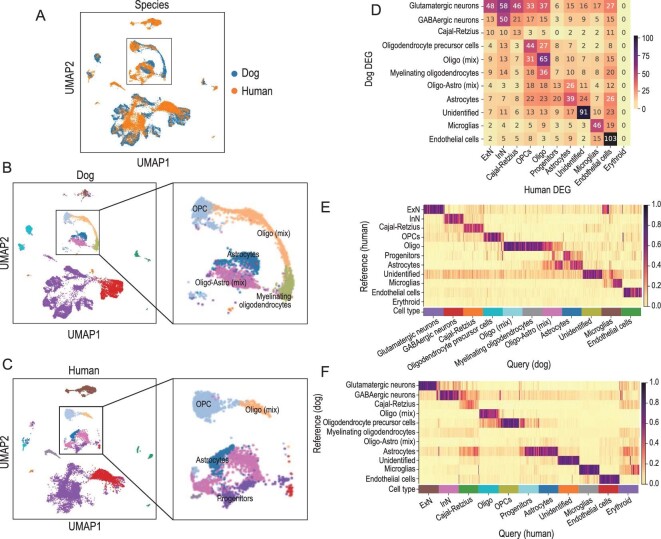
Cross-species comparison between dog and human hippocampal cells. (A)–(C) The UMAP visualization of dog and human hippocampal cells in the integrated space (by Harmony), colored by species (A), the major-type annotations of dog (B) and human (C). (D) The one-to-one homologous overlaps between the DEG sets of dogs and humans. (E) The predicted cell-type probabilities of each cell in the dog hippocampus, output by CAME, using the human data as the reference. (F) The predicted cell-type probabilities of each cell in the human hippocampus, output by CAME, using the dog data as the reference. Oligo-Astro (mix): a mix of oligodendrocytes and astrocytes; Oligo (mix): a mixing of oligodendrocytes; Oligo: oligodendrocytes.

To validate the cell-type annotations and explore the conservation of the hippocampus, we performed a cross-species comparison between dog and human hippocampus using human hippocampus scRNA-seq data from Zhong *et al.* [21]. We used Harmony [31] to integrate these data sets based on the one-to-one homologous genes between dogs and humans. We found that most of the homologous major cell types were consistent and located nearby on the UMAP plots (Fig. 2A–C). The homologous DEG overlaps between each pair of major dog and human types also revealed a similar correspondence (Fig. 2D). In particular, five typical markers are shared between dog and humans (Supplementary Table S4). *GAD1*-expressing inhibitory neurons are associated with human developmental and epileptic encephalopathy [32]. *MBP* marks oligodendrocytes and encodes the protein of the myelin membrane in the CNS [33] and several studies have shown that *MBP* is a biomarker for multiple sclerosis [34]. *AQP4*, an astrocyte marker, plays an important role in brain water homeostasis [35]. *SPARC* inhibits mitogenic effects in microvascular endothelial cells [36]. *PTPRC* (also known as CD45) marks microglia cells [37]. In addition, we applied the cross-species cell-typing and integrative tool CAME [38] to predict the major cell types of dog hippocampal cells (the query) with human snRNA-seq data as the reference (Fig. 2E). We also performed the reverse prediction, i.e. querying the human cells using dog data as the reference (Fig. 2F). We found that the cross-species cell-type predictions of CAME were consistent with the results of Harmony integration and the DEG comparisons. These results suggest that the cell-type annotations of the dog hippocampus are reasonable and that the major types in the hippocampus are highly conserved between dogs and humans.

### Putative trajectory analysis reveals the conserved oligodendrocyte development trajectory

**Figure 3.**
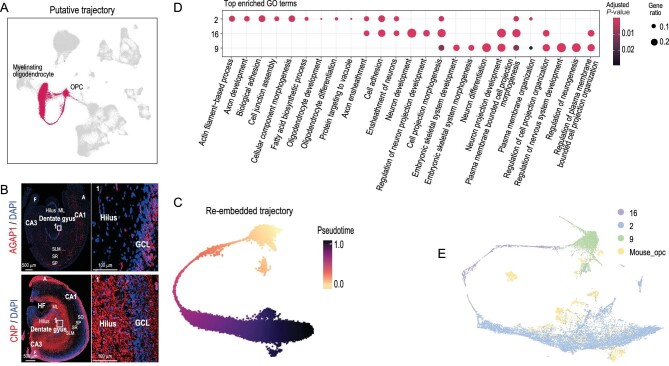
Putative trajectory analysis and cross-species transcriptome comparison between dog and mouse oligodendrocyte. (A) UMAP embedding of oligodendrocyte subclusters. Red indicates cells of Clusters 2, 16 and 9. (B) Staining of *AGAP1* and *CNP* of dog brain at hippocampus. Red: *AGAP1* (top) and *CNP* (bottom), blue: DAPI; white square indicates region boxed had higher-magnification view. (C) UMAP embedding of OPCs putative trajectory. Cells are colored by pseudotime, with dark colors representing mature cell stages and light colors representing immature cell stages. (D) GO enrichment analysis of OPCs differentiation clusters. The color scale represents adjusted *P*-value; dot size represents the gene ratio. (E) UMAP visualization of 20 989 single cells (15 920 from dog and 5069 from mouse) analysed by LIGER, color-coded by cell types.

With the defined cell-type markers, Clusters 2 and 9 were inferred to be myelinating oligodendrocytes and OPCs, respectively (Fig. 3A). Consistently with this classification, *CNP*, a myelin-related marker gene of Cluster 2 [39], was highly expressed in the hilus, ML, SLM, F and A areas (Fig. 3B). *AGAP1* was a DEG in Cluster 9 and could be used as a new marker of OPCs (Fig. 3B). Cluster 16 was linked to Clusters 2 and 9 (Fig. 1A), with few specifically expressed genes to assign its identity. Therefore, we inferred that Clusters 2, 9 and 16 might form a development trajectory from OPCs to myelinating oligodendrocytes, which was also found in the mouse hippocampus [22].

To validate this hypothesis, we constructed the single-nucleus trajectories using Slingshot [40] based on the recalculated UMAP embedding and the original cluster labels (Fig. 3C). As a result, *PTPRZ1*, the marker gene for precursor cells that maintained OPCs in an undifferentiated state [41], was expressed in Cluster 9; *SOX6* was expressed in Cluster 16 and repressed oligodendrocyte differentiation [42]; *PLP1* was another myelin-related gene expressed in Cluster 2 (Supplementary Fig. S2A). The GO enrichment analysis showed that oligodendrocytes (Clusters 2, 9 and 16) were involved in different biological pathways (Fig. 3D). The DEGs in Cluster 9 (OPCs) were related to neuron differentiation (GO : 0030182, Supplementary Table S5) and regulation of neurogenesis (GO : 0050767, Supplementary Table S5). Those in Cluster 16 were related to cell projection morphogenesis (GO : 0048858, Supplementary Table S5) and neuron projection development (GO : 0031175, Supplementary Table S5). Those in Cluster 2 were enriched in ensheathment of neurons (GO : 0007272, Supplementary Table S5) and axon ensheathment (GO : 0008366, Supplementary Table S5). The DEGs of the putative trajectory were consistent with the differentiation process of oligodendrocyte development and we also verified this result in mouse data (Fig. 3E and Supplementary Fig. S2B–E). Additionally, we combined the data with single-cell transcriptomes from the human hippocampus for comparative analysis and validation [21]. The results showed that the hippocampus followed a developmental trajectory from OPCs to oligodendrocytes in humans and dog (Fig. 2A–C). All these results indicate that humans, dog and mice have a similar trajectory of oligodendrocyte development.

### Significant convergence between DEGs and putative PSGs in domestication

Previous studies showed that dog putative PSGs were enriched in neurological processes such as learning and memory [5,43] and expressed specifically in brain tissues [14]. To identify which types of cells play an important role during domestication, we gathered the DEGs in the 26 clusters (with *P*-value < 0.001 and log_2_(fold change) > 0.25) and compared them with the putative PSGs from published studies [5,13,43–45]. The genes expressed in more than three cells in the dog hippocampus were used as the background (see ‘Methods’ section). The results showed that 630 of 841 putative PSGs were detected in the hippocampus single-nucleus transcriptomes (Supplementary Table S6 and Supplementary Fig. S3). Eighty-six of 630 detected putative PSGs occurred in the 1628 DEGs with a statistical significance with a *P*-value of 5.10E-09 (Table 1). However, the 1000 random gene sets with equal sizes were not significant, with a *P*-value of 0.47. Since Freedman *et al.* (2016) performed a statistical analysis to determine the likelihood that specific genes were under selection, we used their putative PSGs to verify the statistical significance. As a result, 16 genes occurred in both DEGs and putative PSGs with a *P*-value of 6.99E-03. Furthermore, there was also a significant overlap between DEGs and the putative PSGs reported in at least two published studies (*P*-value = 2.45E-07, Supplementary Table S7).

**Table 1.** Overlapped genes between putative PSGs and DEGs.

Cell type	Cluster	Gene
Glutamatergic neurons	0, 3, 5, 6, 7, 10, 11, 13, 14, 15, 17, 20	*ADCY8, ARHGAP32, ASAP1* ^a^, *CACNA1A, CADM2*^a^, *CADPS2*^a^, *CCBE1, CNTN5, CUX2*^a^, *DGKI, EEF1A1*^a^, *EML6, FOXP2*^a^, *FSTL4, GLIS1, GRIK3, HAPLN4, KCNJ3, KCNMA1*^a^, *KLF12*^a^, *LRRTM3*^a^, *NRXN3*^a^, *NTNG1, PIK3R1, PPM1E, PRKCA*^a^, *PRRC2B, RFX3*^a^, *RIMS2*^a^, *RYR3, SATB2, SBF2, SCN2A*, SYN2^a^, *TCF4*^a^, *TIAM1, TMEM132D*^a^, *TMEM59L, YWHAH, ZMAT4*^a^
GABAergic neuron	4, 8, 12, 21	*CADPS2, CUX2, GABRA4, GAD2, KCNC2* ^a^, *NRXN3, PRKCA, ROR1, SYN2, TCF4, ZMAT4*
Oligodendrocytes	2, 9, 16, 18	*AGAP1, ANKRD44* ^a^, *ASAP1, BAZ2B*^a^, *CADM2, CCSER2, CLIC4, COL11A1, ENSCAFG00000031463, FAM107B, HIPK2*^a^, *ITCH, KLF12, LIMCH1, LRRTM3, MAP3K1, MBP, PLXDC2*^a^, *PRKCA, RALGAPA2*^a^, *REEP3, RIMS2, SAMD12, TMEM132D*
Microglia	24	*ANKRD44, BAZ2B, DOCK2, ENTPD1, IKZF1, KLF12, MRC2, PLXDC2, SHB, SLCO2B1* ^a^, *SSH2, TBXAS1*
Other	1, 19, 22, 23, 25	*AHCYL2, ATXN2, CAPS2, CUX2, DNAH3, EEF1A1, FHOD3, FOXP2, GNA12, HIPK2, HYDIN, ITGB1, KCNC2, KCNMA1, KIAA1328, MYOF, PDE7B, PPM1H, PSD2, RALGAPA2, RBPMS, RFTN2, RFX3, SLCO2B1, TOM1L2, WDR66*

^a^Indicates this gene appears in different cell types, i.e. *CUX2* in Glutamatergic neurons, GABAergic neurons and non-neurons.

To explore whether putative PSGs have higher or lower specificity across clusters or cell types, we calculated the entropy of putative PSGs. The higher entropy-specificity score for a gene implies its uniqueness in specific clusters or cell types [46]. We found that the putative PSGs that occurred in more than one study have significantly higher entropy specificity across both clusters and cell types compared with the background (Table 2). To determine which cluster was highly enriched with putative PSGs, we performed enrichment analysis for each cluster-specific in the putative PSG set (Table 3). Four clusters (Clusters 2, 9, 14 and 24) were significantly enriched in three putative PSG sets and six clusters (Clusters 0, 8, 11, 16, 17 and 20) were enriched in two putative PSG sets (with *P*-value < 0.05).

**Table 2.** The entropy of putative PSGs in different cell types and cluster.

		Gene-specificity based on the average expressions of cluster			Gene-specificity based on the average expressions in a cell type		
	Proportion-cut	PSG-841	PSG-78	PSG-145	PSG-841	PSG-78	PSG-145
PSG	0	1.57E-01	1.46E-01	1.85E-01	2.11E-01	1.94E-01	2.36E-01
bg	0	2.47E-01	2.45E-01	2.45E-01	3.31E-01	3.28E-01	3.28E-01
PSG	0.1	7.96E-02	1.26E-01	1.05E-01	1.01E-01	1.52E-01	1.27E-01
bg	0.1	9.36E-02	9.28E-02	9.29E-02	1.20E-01	1.19E-01	1.19E-01
PSG	0.2	7.43E-02	1.23E-01	8.97E-02	9.21E-02	1.52E-01	1.10E-01
bg	0.2	8.97E-02	8.87E-02	8.89E-02	1.14E-01	1.13E-01	1.13E-01
PSG	0.25	7.00E-02	1.07E-01	9.32E-02	8.68E-02	1.34E-01	1.15E-01
bg	0.25	9.00E-02	8.88E-02	8.89E-02	1.14E-01	1.13E-01	1.13E-01

bg, background genes that were expressed by at least a proportion of ‘proportion-cut’ in any cell cluster to filter out those genes having low expression frequencies; PSG-841, putative PSGs in five articles; PSG-78, putative PSGs occurred in no fewer than two articles; PS-145, putative PSGs occurred in Freedman *et al.* 2016.

**Table 3.** Enrichment analysis for each cluster-specific genes in putative PSG sets.

Cluster	PSG-841	PSG-78	PSG-145
0 Glutamatergic neurons	4.06E-05	6.18E-03	1.42E-01
0 Glutamatergic neurons (random)	4.33E-01	2.32E-01	3.00E-01
3 Glutamatergic neurons	2.38E-03	5.35E-02	4.86E-01
3 Glutamatergic neurons (random)	4.30E-01	2.25E-01	3.04E-01
5 Glutamatergic neurons	4.37E-01	3.76E-01	2.04E-01
5 Glutamatergic neurons (random)	4.27E-01	2.63E-01	3.26E-01
6 Glutamatergic neurons	9.51E-02	2.31E-01	3.75E-01
6 Glutamatergic neurons (random)	4.01E-01	1.87E-01	2.63E-01
7 Glutamatergic neurons	1.38E-01	2.57E-01	4.11E-01
7 Glutamatergic neurons (random)	4.06E-01	1.94E-01	2.66E-01
10 Glutamatergic neurons	1.02E-02	2.77E-01	4.39E-01
10 Glutamatergic neurons (random)	4.10E-01	2.14E-01	2.89E-01
11 Glutamatergic neurons	7.28E-05	2.43E-02	6.88E-02
11 Glutamatergic neurons (random)	4.22E-01	1.74E-01	2.43E-01
13 Glutamatergic neurons	1.83E-01	2.15E-01	3.50E-01
13 Glutamatergic neurons (random)	4.08E-01	1.74E-01	2.52E-01
14 Glutamatergic neurons	1.77E-02	2.04E-04	1.51E-02
14 Glutamatergic neurons (random)	4.04E-01	1.99E-01	2.69E-01
15 Glutamatergic neurons	5.16E-02	2.52E-01	4.05E-01
15 Glutamatergic neurons (random)	4.11E-01	1.95E-01	2.79E-01
17 Glutamatergic neurons	1.54E-03	3.47E-02	4.08E-01
17 Glutamatergic neurons (random)	4.05E-01	1.98E-01	2.81E-01
20 Glutamatergic neurons	8.16E-02	4.23E-02	2.06E-02
20 Glutamatergic neurons (random)	4.33E-01	2.12E-01	2.79E-01
4 GABAergic neurons	7.88E-02	4.18E-03	1.13E-01
4 GABAergic neurons (random)	4.05E-01	2.14E-01	2.91E-01
8 GABAergic neurons	2.32E-03	2.09E-03	3.64E-01
8 GABAergic neurons (random)	3.99E-01	1.80E-01	2.53E-01
12 GABAergic neurons	2.08E-01	4.87E-03	4.58E-01
12 GABAergic neurons (random)	4.18E-01	2.21E-01	2.99E-01
Cluster	PSG-841	PSG-78	PSG-145
21 GABAergic neurons	1.57E-01	5.90E-02	5.06E-01
21 GABAergic neurons (random)	4.27E-01	2.39E-01	3.05E-01
25 Cajal-Retzius	2.69E-01	1.78E-01	2.95E-01
25 Cajal-Retzius (random)	3.79E-01	1.48E-01	2.19E-01
1 Astrocytes	1.46E-01	1.07E-01	2.62E-01
1 Astrocytes (random)	4.26E-01	2.81E-01	3.50E-01
24 Microglia	1.09E-05	3.34E-04	3.11E-04
24 Microglia (random)	4.15E-01	2.13E-01	2.82E-01
9 Oligodendrocyte precursor cells	2.38E-03	5.30E-04	5.45E-04
9 Oligodendrocyte precursor cells (random)	4.42E-01	2.21E-01	3.07E-01
16 Unknown	8.92E-02	2.72E-03	3.84E-03
16 Unknown (random)	4.42E-01	2.84E-01	3.55E-01
2 Myelinating oligodendrocytes	6.22E-03	2.57E-02	5.62E-03
2 Myelinating oligodendrocytes (random)	4.42E-01	2.96E-01	3.53E-01
18 Myelinating oligodendrocytes	3.81E-01	4.52E-02	1.22E-01
18 Myelinating oligodendrocytes (random)	4.05E-01	2.18E-01	2.88E-01
19 Non-neuron	1.46E-01	2.37E-03	2.62E-01
19 Non-neuron (random)	4.45E-01	2.87E-01	3.46E-01
22 Non-neuron	2.12E-01	1.59E-01	7.21E-01
22 Non-neuron (random)	4.43E-01	3.11E-01	3.71E-01
23 Endothelial cells	2.28E-01	1.01E-01	7.12E-02
23 Endothelial cells (random)	4.26E-01	2.77E-01	3.45E-01

PSG-841, putative PSGs in five articles; PSG-78, putative PSGs occurred in no fewer than two articles; PSG-145, putative PSGs occurred in Freedman *et al.* 2016.

**Figure 4.**
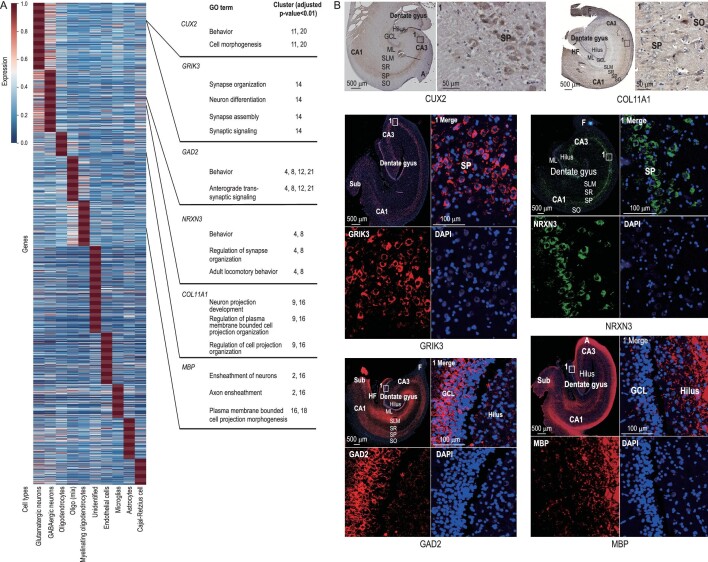
The mean expression of DEGs in different cell types. (A) The values of each gene (row) were its original values divided by its maximum (Supplementary Table S13). (B) Six genes in Fig. 4A expression in the dog hippocampus revealed by immunohistochemical and immunofluorescent staining. Six genes on the right of the picture were differentially expressed in different cell types and the GO enrichment analysis showed their relevant gene functions. It is worth noting that *CUX2* and *GRIK3* are DEGs in glutamatergic neurons, although compared to other cells they are highly expressed in non-neurons and GABAergic neurons, respectively. High-definition immunohistochemical and immunofluorescent staining is shown in Supplementary Fig. S4A.

Glutamatergic receptors constitute a major excitatory transmitter system, and dog excitatory synaptic plasticity increased in the domestication process [15]. Forty of 86 putative PSGs were expressed in glutamatergic neurons and 28 genes were expressed in Clusters 0, 11, 14, 17 and 20. The GO enrichment analysis showed that DEGs of Cluster 0 were enriched in the regulation of cell migration (GO : 0030334, Supplementary Table S2) and regulation of cell motility (GO : 2000145, Supplementary Table S2), those in Clusters 11 and 14 were involved in neuron differentiation (GO : 0030182, Supplementary Table S2) and those in Clusters 17 and 20 were enriched in axon guidance (GO : 0007411, Supplementary Table S2) and neuron projection guidance (GO : 0097485, Supplementary Table S2). A putative PSG *CUX2* was highly expressed in DG granular cells as glutamatergic neuron DEGs in Clusters 11 and 20, and it was also expressed in CA granular cells, as the same expression pattern in mice (Fig. 4A and B and Supplementary Fig. S4B) [47]. Another putative PSG, *GRIK3*, in Cluster 14 was detected mainly in the GCL of the DG and SP of the CA area (Fig. 4A and B). It is worth noting that the putative PSGs were significantly enriched in Cluster 14 (Table 3), and the DEGs in Cluster 14 were enriched in synapse organization (GO : 0050808, Supplementary Table S2), neuron differentiation (GO : 0030182, Supplementary Table S2), synaptic signaling (GO : 0099536, Supplementary Table S2) and synapse assembly (GO : 0007416, Supplementary Table S2).

Eleven putative PSGs were expressed in GABAergic neurons, seven of them belonged to Cluster 8. GO enrichment analysis suggested that DEGs in Cluster 8 were involved in behavior (GO : 0007610, Supplementary Table S3), forebrain development (GO : 0030900, Supplementary Table S3) and adult locomotory behavior (GO : 0008344, Supplementary Table S3). *GAD2* was expressed in Clusters 4, 8, 12 and 21 (Fig. 1E) and it has been taken as a candidate gene to regulate brain development and behavior during domestication (Fig. 4A and B) [13]. Moreover, DEGs in Clusters 4, 8, 12 and 21 were enriched with behavior (GO : 0007610, Supplementary Table S3) and anterograde transsynaptic signaling (GO : 0098916, Supplementary Table S3). Previous studies suggested that ablation of *GAD2* in mice reduced freezing and increased flight and escape behavior [48]. *NRXN3* (Clusters 4 and 8) encodes a receptor and cell adhesion molecule in the nervous system, and it was highly expressed in the SP of the hippocampal CA area (Fig. 4A and B). GO enrichment analysis showed that DEGs in Clusters 4 and 8 were relevant to behavior (GO : 0007610, Supplementary Table S3), regulation of synapse organization (GO : 0050807, Supplementary Table S3) and adult locomotory behavior (GO : 0008344, Supplementary Table S3). A previous study also confirmed that putative PSGs in Chinese native dogs were enriched in locomotory behavior [14].

Twenty-four putative PSGs occurred in oligodendrocytes, including Clusters 2, 9, 16 and 18. Three of them are related to OPC differentiation. Oligodendrocytes are a kind of cell that primarily forms CNS myelin [49]. One of the overlapping genes, *COL11A1* (Clusters 9 and 16), marked OPCs and was expressed in the partial area of the hippocampal CA (Fig. 4A and B). Previous studies have shown that OPCs are important for human white matter expansion and myelination [50]. Myelinating oligodendrocytes were the result of OPC differentiation. *MBP* was a marker gene that was expressed in Clusters 2, 16 and 18, and highly expressed in the hilus, ML, SLM and other hippocampal regions (Fig. 4A and B). As a domestication gene, *MBP* encodes myelin basic protein. Domestication influenced myelination and/or axonal diameter in rabbits [51] and affected neurological function in dogs [43]. The relevant functions in these clusters were ensheathment of neurons (Clusters 2 and 16, GO : 0007272, Supplementary Table S8) and axon ensheathment (Clusters 2 and 16, GO : 0008366, Supplementary Table S8).

### Reconstructed gene regulatory networks of the dog hippocampus

We reconstructed dog hippocampal gene regulatory networks using GENIE3 software [52]. To attenuate the effects of noise and outliers, we used 4523 genes and 4281 pseudo cells (see ‘Methods’ section about Weighted correlation network analysis, WGCNA), which contained all the cell types in this study. More than 2 million interactions were found between the putative regulatory genes and target genes, with 2239 putative regulatory genes in the top 1% (Supplementary Table S9). The transcription factors in dogs (*Canis familiaris*) from AnimalTFDB3.0 [53] were used to verify these putative regulatory genes. As the results showed, there were 118 putative regulatory genes, 11 of which were both PSGs and DEGs, including Cut Like Homeobox 2 (*CUX2*) [13,43] and Regulatory Factor X3 (*RFX3*) [5], in glutamatergic neurons. GO enrichment analysis showed that the target genes of *CUX2* were related to glutamatergic synaptic transmission (GO : 0035249, Supplementary Table S10). The transcription factor CUX2 is involved in early neocortical circuits, cellular fate selection and mechanosensation development [54,55]. Another regulatory gene, *RFX3*, is expressed in the DG in mice [56] and its target gene was enriched in neurogenesis (GO : 0022008, Supplementary Table S11), neuron development (GO : 0048666, Supplementary Table S11) and locomotion (GO : 0040011, Supplementary Table S11). Taken together, the results above implied that glutamatergic neurons may be involved in the adaptive evolution of dog behaviors.

## DISCUSSION

In this study, we presented single-nucleus transcriptomics of the dog hippocampus and identified 26 cell clusters based on 105 057 single-nucleus transcriptomes, including glutamatergic neurons, GABAergic neurons, Cajal-Retzius cells, OPCs, myelinating oligodendrocytes, endothelial cells, astrocytes, microglia cells and so on. In addition, our study demonstrated the trajectory of oligodendrocyte differentiation based on the recalculated UMAP embedding analysis. The cross-species analysis revealed that eight major cell types were shared between the human and dog hippocampus [21], suggesting that dogs have potential as a model organism for human mental illness.

Gene expression has spatial heterogeneity, which could reveal the relationship between hippocampal subareas and cell clusters. The hippocampus is divided into two main parts, namely the cornu ammonis and the DG, which contain pyramidal neurons and granule cells, respectively [57]. Clusters 19 (unidentified), 20 (glutamatergic neurons), 25 (Cajal-Retzius cells) and 16 (oligo-mix) might be distributed in the DG because these clusters had the highest expression proportion and average expression of *CUX2, CXCR4* and *SEMA5A*, respectively (Supplementary Table S12). Immunohistochemical showed that *CUX2* was highly expressed in the DG (Fig. 4B and Supplementary Fig. S4A), and *CXCR4* and *SEMA5A* are also expressed in the DG [21,58]. Clusters 7 (glutamatergic neurons) and 20 (glutamatergic neurons) might be located in CA3 since these two clusters had the highest expression proportion and average expression in *NRIP3* and *SULF2*, respectively. These two genes are marker genes for CA3 [21]. Cluster 19 might also be located in CA1 because *PID1*, a marker gene for CA1, had the highest expression proportion and average expression [21]. Clusters 6 (glutamatergic neurons) and 13 (glutamatergic neurons) had the highest expression proportion and average expression in *RGS14*, which is a CA2 marker; therefore, we inferred that these two clusters might be located in CA2 [59].

Reduced fear and aggression are important traits selected by humans in the first step of animal domestication, which help animals not only live commensally with humans, but also stay in a crowded environment [60,61]. As a subtype of inhibitory neurons, GABAergic neurons have been linked to the response to learning and fear memory [62,63]. Domestication may reduce myelination levels, compromise neural conduction, also change the size of brain structures relevant for memory, reflexes and fear processing [51]. The lower reactive level is a component of domestication syndrome, which is a collection of common traits in domestic animals [64]. In our data, the putative PSGs in domestication were involved in the development of oligodendrocytes and might be involved in myelination levels during nervous system development.

Glutamate receptors play an important role in the CNS and respond to basal excitatory synaptic transmission; some of them are involved in learning and memory, and there were great changes in glutamate receptors in animal domestication and modern human evolution [65]. Furthermore, Cluster 14 consisted of glutamatergic neurons, and DEGs in the cluster were enriched in the regulation of synapse structure or activity (GO : 0050803, Supplementary Table S2) and regulation of nervous system development (GO : 0051960, Supplementary Table S2). These findings reveal that cells in Cluster 14 may play an important role in a decreased stress response not only in domesticated animals, but also in humans [65]. These results could verify the hypothesis that glutamatergic neurons changed their behavior during domestication and reduced the fear response in dogs by regulating cells in the hippocampus, and Cluster 14 is probably an important cluster in dog domestication.

In summary, 105 057 single-nucleus transcriptomes were classified into 26 clusters in this study and were defined with distinct identities. Further exploration revealed 86 genes that overlapped between putative PSGs and DEGs. In addition, we illustrated the OPC differentiation trajectory based on the UMAP embedding. Our results contributed to defining the cell types and revealing the development of oligodendrocytes in the dog hippocampus, illustrating the difference between the subcell types and connecting the gap in our understanding between the molecular and cellular mechanisms of animal domestication.

## MATERIALS AND METHODS

Information on the materials used to conduct the research and descriptions of all methods used in the analysis are available in the Supplementary information. A 5-month-old Beagle was used in this work; the dog was provided by the Department of Laboratory Animal Science, Kunming Medical University. All animal processing procedures and experiments performed in the present study were approved by the Animal Ethics Committee of Kunming Institute of Zoology, Chinese Academy of Sciences (SMKX-20 160 301-01).

## DATA AVAILABILITY

The data underlying this article are available in the Genome Sequence Archive in the BIG Data Center, Beijing Institute of Genomics (BIG), Chinese Academy of Sciences, and can be accessed with PRJCA004294. We also constructed the dog hippocampus atlas website at https://ngdc.cncb.ac.cn/idog/ (single-cell module).

## Supplementary Material

nwac147_Supplemental_Files
