# Supplementary material for: A single-nucleus transcriptomic atlas of the dog hippocampus reveals the potential relationship between specific cell types and domestication
Source: Natl Sci Rev. 2022 Jul 23;9(11):nwac147. doi: 10.1093/nsr/nwac147 (PMC9772819; doi:10.1093/nsr/nwac147)
Supplement: nwac147_Supplemental_Files [file nwac147_supplemental_files.zip › Supplementary_Information.docx]

**Supplementary Information**

**Supplementary Information-** **Materials and Methods**

**Figure S1 Clustering analysis detail information.**

**Figure S2 Gene expression patterns of oligodendrocytes and molecular diversity between dog and mice.**

**Figure S3 The mean expression of 630 putative PSGs in different cell types.**

**Figure S4 High-definition immunohistochemical staining.**

**Table S1 DEG list.**

**Table S2 DEGs in glutamatergic neurons: GO enrichment analysis.**

**Table S3 DEGs in GABAergic neurons: GO enrichment analysis.**

**Table S4 Shared DEGs between dogs and humans**

**Table S5 DEGs in OPC differentiation: GO enrichment analysis.**

**Table S6 Expression of 630 PSGs in each cell type.**

**Table S7 Putative PSGs and DEGs: statistical analysis.**

**Table S8 DEGs in Clusters 2, 16, and 18: GO enrichment analysis.**

**Table S9 Interaction according to GENIE3.**

**Table S10 GO enrichment analysis results for the regulatory gene *CUX2*.**

**Table S11 GO enrichment analysis result for the regulatory gene *RFX3*.**

**Table S12 Gene expression average and proportion.**

**Table S13 The mean expression of DEGs in different cell types.**

**Table S14 Three differential expression analyses.**

**Table S15 Domestication-related gene list.**

**Table S16 Antibody used in IF and IHC of the *Canis* hippocampus.**

**Supplementary Information**

**Materials and Methods**

*Tissue dissection and nucleus extraction*

Broader dissections (no layer enrichment or multiple layers combined) were used to facilitate isolation of a sufficient number of cells. The nucleus extraction protocol was adapted from Rosenberg et al. (2018) [1]. Briefly, a dounce homogenizer (Wheaton, cat. no. 357538) was used for nucleus extraction. Hippocampus was homogenized in the dounce homogenizer (Kimble) 10X with lose pestle and 20X with tight pestle. A homogenization buffer (4.845 mL of NIM1 buffer (250 mM sucrose, 25 mM KCl, 5 mM MgCl2, 10 mM Tris pH=8.0), 5 μL of 1 mM DTT, 50 μL of Enzymatics RNase Inibitor (40U/μL), 50 μL of SuperaseIn RNase Inhibtor (20U/μL), 50 μL of 10% Triton-X100) was used to make a homogeneous nucleus solution and protect the RNA from degradation. Nuclei were collected by the centrifugation and filtered through sterile 40μm cell strainer (Corning) into 1.5ml tubes. The nucleus concentration was checked by the hemocytometer to ensure it was within 1,000,000 nuclei/ml.

*Library preparation*

SPLiT-seq (split-pool ligation-based transcriptome sequencing) was used to generate the libraries with Uniquely Barcoded Cells (UBC) [1]. Briefly, nuclei were distributed into 48 individual wells in two 96-well plates. Each well loaded about 5,000-8,000 nuclei. The random hexamer and the anchored poly(dT)15 barcoded RT primers were respectively distributed into 48 individual wells with nuclei of the 96-well. The links between well ID and barcode were recorded for downstream data processing. For the other protocol of the reverse transcription, ligation barcoding, lysis, cDNA amplification, tagmentation and Illumina amplicon generation were processed according to the SPLiT-seq protocol Version 3.0. The libraries were processed on the Illumina platform for sequencing of 150 bp pair-end reads.

*Processing of raw scRNA-seq data*

According to the procedure of SPLiT-seq, the site of read 11-18 bp that did not match the barcode list were removed. The sequencing data was processed with the Drop-seq core computational protocol ("http://mccarrolllab.org/dropseq/") to align, filter, and count unique molecular identifiers (UMIs) per sample by default. Data were mapped to the dog reference genome CanFam3.1 (GCA_000002285.2) and the transcriptome annotation from the Ensembl database, Canis_familiaris. CanFam3.1.92. Cells with more than 350 and no more than 5,000 expressed genes were retained. After that, genes expressed in less than three cells were filtered out. At the same time, cells that were expressed in a high proportion of mitochondrial genes (> 0.03) were also removed. As a result, 18,039 cells from the random group and 87,018 cells from the poly-T group were used for further analysis.

*Data normalization and HVG selection*

The gene-by-cell matrices were adjusted by a total-count normalization. Formally, denote $x_{ij}$ as the raw count of gene $i$ in cell $j$. For each cell $j$, counts were divided by its total count $c_{j}=\sum_{i} x_{ij}$ of this cell and then multiplied by the median of all the total counts $m=median\{c_{j}\}$. The total-size normalized expressions $\hat{x}_{ij}=mx_{ij}/c_{j}$ were then log-transformed to $\hat{x}_{ij}\leftarrow log(\hat{x}_{ij}+1)$ for downstream analysis. Highly variable genes (HVGs) were selected separately within random-group and poly-T-group, using the build-in function “scanpy.pp.highly_variable_genes” of the Python package ScanPy [2]. Genes with a (log-normalized) mean expression above 0.025 and a normalized dispersion higher than 0.25 were identified as highly variable ones in each group. Finally, we took those genes that were highly variable in both groups and those with top dispersion as final HVGs for downstream analysis. This process resulted in 2,000 HVGs for downstream analysis.

*Dimensionality reduction and clustering*

Before performing dimensionality reduction, the gene-by-cell expression matrix was centralized and scaled within each group of the same RNA capturing primer, for elimination of batch effect. The partial-PCA was performed to combine the random and poly-T groups. We calculating the principal components on the polyT-group first, and projected the random-group onto the same PC space of the polyT-group. We selected the top 50 principal components (PCs) with the highest explained variances. We also tried different numbers of PCs and got similar results. UMAP [3] was used to embed each cell from the reduced PC space into a 2D space. It first computed the approximate k nearest neighbors (KNNs) for each data point, built a weighted mutual-KNN graph with each node representing each cell, and embedded each node of the graph into the low-dimensional space. We computed 30 approximate nearest neighbors for each single cell based on cosine distance in the PC space. Leiden community detection algorithm [4] was applied (with resolution=0.8, which resulted in a fine grained clustering) onto the weighted KNN graph built by UMAP to cluster single cells into distinct groups.

*Identification of DEGs and enrichment analysis*

We compared the transcriptomic profile of each cluster versus the others using three differential analysis methods to get reliable DEG sets (Student’s t-test, Wilcox [5], MAST [6]) with the same thresholds (p-values < 0.001 and log2fold-changes > 0.25). The intersection of them were 64 to 233 DEGs for each cluster (**Table S14**). Each detected cluster was mapped to cell types or intermediate states by matching their corresponding DEGs to Allen Brain Atlas database and consulting published literatures. GO enrichment analysis of these DEGs was performed by the R package “clusterProfiler” [7] with the reference database “org.Cf.eg.db” [8]. Instead of the genes in the whole genome of dog, we used the genes that were detected in the present hippocampus data as the background.

*Categorization of genes using WGCNA*

We adopted WGCNA (Weighted gene correlation network analysis) [9] analysis to detect gene modules with the R package “WGCNA” (R version 3.6.3, https://cran.r-project.org/web/packages/WGCNA; package version 1.69), which was initially designed for the bulk RNA-seq data. For the convenience of calculation, we used 4,523 genes expressed in more than 25% of populations in at least one cluster. Considering the large number of single-nucleus and the sparsity of the expression profiles, samples used in the WGCNA were created by aggregating and averaging each of the small clusters (< 100 cells) of single cells of similar transcripts, resulting 4,281 pseudo cells. The soft power value (power = 4) was determined by inspecting the soft-threshold-mean-connectivity curve plot. Modules with distance less than 0.25 (i.e., correlation more than 0.75) were merged. The module-cluster relationships were evaluated with the Pearson correlation coefficients between the module-memberships (MMs, a gene-by-module matrix) for genes and the gene-significances (GSs, a gene-by-cluster matrix) for clusters. The MM was defined as the Pearson correlation coefficients between the pseudo cell expressions and the eigengenes of the modules (computed by the WGCNA), while the GS was defined as the Pearson correlation coefficients between the pseudo cell expressions and the one-hot coded cluster labels.

*Cross-species analysis between dog and human hippocampus*

We adopted the scRNA-seq data of human hippocampus from Zhong et al. (2020) [10]and reanalyzed and annotated the cells according to the markers reported in this study (since the authors did not provide the cell-type annotations). To get the cell-type specific genes (i.e., the DEGs), we perform differential analysis on the normalized human hippocampus data using the Seurat function ‘FindAllMarkers’ with the parameter ‘test.use’ setting as “MAST”. The genes with adjusted p-value higher than 0.001 was filtered out. Then we performed a cross-species comparison of the DEGs between each cell type in human and dog hippocampus, based on the one-to-one homologous gene mappings downloaded from BioMart (Ensembl 106) [11]. The number of cells of dog hippocampal snRNA-seq is about three times of that of human data and the cell compositions are quite imbalanced, which might make the small groups being covered after integration. Considering this, we performed a group-balanced subsampling for both dog and human transcriptomic data. For each cell population, if it contained more than 1,000 cells, 1,000 cells were randomly sampled and kept. This resulted in 22,363 cells and 16,320 cells in dog and human data respectively. To get the integrated visualization of dog and human hippocampus cells, we performed Harmony integration on the PCA embeddings of the merged datasets with only one-to-one homologous genes. And the batch-corrected embeddings were used for computing the UMAP coordinates. To make a cross-species cell-type mapping, we applied a cross-species cell-typing and integrative tool CAME [12] to predict the major cell types of human hippocampal cells (the query) with the dog snRNA-seq data as the reference, and also query the dog cells using human data as the reference.

*Putative trajectory analysis for a subset of hippocampus cells*

To validate our hypothesis of the trajectory, we separated these clusters of cells, and redid dimensionality reduction using PCA and UMAP. Moreover, we analyzed the polyT group only for those holding the majority (15,791 cells, nearly 90% of the entire trajectory) of the transcriptomes that formed the trajectory. Therefore, we can keep the biological information from being lost without introducing additional technical noise. In detail, we selected the top 20 PCs according to the PC-elbow plot, found 10 nearest neighbors for each cell using Euclidean distance and performed UMAP with the parameter “min_dist = 0.2”. After that, the pseudo-time of each cell on the trajectory was inferred using Slingshot, based on the re-calculated UMAP embeddings and the original cluster labels. The student’s t-test was performed to find out the DEGs within the three clusters that formed the trajectory.

*Cross-species integration of oligodendrocyte between dog and mouse*

Integration of single-cell data from mouse and domestic dogs was processed with LIGER (liger) [13], which is an R package for integrating and analyzing multiple single-cell datasets. We downloaded 5,072 transcriptomes of single cells from the mouse oligodendrocyte lineage obtained at GEO (GSE75330) and separated cells of correlative clusters to do integrative analysis. We used LIGER with the following processing and parameters. First, we normalized the data to account for differences in sequencing depth and capture efficiency across cells, selected variable genes, and scaled the data with “var.thresh = 0.1”. Next, we performed integrative non-negative matrix factorization to identify shared and distinct metagenes across the datasets using the LIGER function “optimizeALS (k = 25)”. We performed a quantile alignment step “quantileAlignSNF” with the default settings and used UMAP to visualize the integrated data.

*Enrichment analysis of DEGs on putative PSG sets*

The domesticated gene list was obtained from five studies (**Table S15**) [14-18]. We pooled the DEGs from all clusters for the enrichment analysis based on the hypergeometric test. The hypergeometric distribution, which describes the probability of k successes (random draws for which the object drawn has a specified feature) in n draws, without replacement, from a finite population of size N that contains exactly K objects with that feature, where each draw is either a success or a failure. The probability distribution function is given by$P\left( X=k \right)=\frac{\left( \begin{aligned} K \\ k \end{aligned} \right)\left( \begin{aligned} N-K \\ n-k \end{aligned} \right)}{\left( \begin{aligned} N \\ n \end{aligned} \right)}$, where $\left( \begin{aligned} n \\ m \end{aligned} \right)$ is the binomial coefficient. And the probability of over-representation is $\sum_{k\leq i\leq n} P\left( X=i \right)$. In our case, we take a total of N=22,639 genes expressed in more than three cells as the gene universe instead of using the whole genome of the dog. K is the number of intersects between a given putative PSG set and the gene universe.

It is worth noting that the PSGs from the published studies were described as putative PSGs in the present study. The four studies of Wang et al., vonHoldt et al., Axelsson et al., and Pendleton et al. reported 205, 29, 122 and 429 PSGs through the whole genome scan for selection, respectively [14, 16-18]. None of those were validated as the PSGs by additional statistical analysis nor the experimental assays. Freedman et al. reported 145 PSGs and performed the statistical analysis to determine the likelihood under selection [15]. Thus, we performed statistical analysis using the putative PSGs in Freedman et al 2016 only, and those reported at least in the two references, leading to the same significant overlap.

*Entropy specificities of putative PSG sets across clusters and cell-types*

We followed [19] and computed the entropy specificity for each gene, based on the normalized average expressions, grouped by those 26 clusters and 10 major cell types. The computation was done by the build-in function ‘entropySpecificity’ of BioQC [20]. To avoid the noises from the lowly expressed genes, we took the genes expressed in more than 10% of populations in at least one cluster as the background. The entropy-specificity scores were normalized and averaged for each putative PSG set and the backgrounds.

*Gene regulatory networks (GRN) analysis by the GENIE3*

Inferable regulatory links for each gene were predicted by the GENIE3 software (version 1.16.0[21] with the random forest machine learning algorithm. The links ranked by weight and only top 1% regulatory genes were reserved for subsequent analysis. Transcription factors of the dog were downloaded from the AnimalTFDB 3.0 [22]. GO enrichment analysis for target genes was performed using the g:Profiler (version e104_eg51_p15_3922dba) by the dog annotation [23].

*Immunofluorescent (IF) and immunohistochemistry (IHC) staining*

Formalin-fixed and paraffin-embedded hippocampus tissue specimens were sliced into 9 µm sections and then deparaffinized in turpentine oil (TO), rehydrated through graded ethanol solutions and antigen retrieval was performed by placing the slides at 95°C for 40 min in a microwave oven and allowed to cool at room temperature. The slides were washed three times with PBS, and then the slides were permeabilized with 1% Triton X solution for 10 min. After nonspecific binding was blocked by 5% Bovine serum albumin (BSA) for 1h at room temperature, the slides were incubated with primary antibodies overnight at 4 °C. For immunofluorescent staining, DAPI (4-6-diamidino-2-phenylindole) and DyLight 488- or DyLight 555- labeled secondary antibodies (1:400, Thermo Fisher Scientific, Waltham, MA) were added for 1.5h at room temperature. For immunohistochemistry staining, the slides were treated with HRP-labeled second antibody (Thermo Fisher Scientific) for 30 min. The slides were incubated with DAB until desired stain intensity is observed and then followed by slight hematoxylin counterstaining. The slides were finally dehydrated and mounted with a cover glass. Stained slides were visualized and imaged using a laser scanning confocal microscope (Olympus, Tokyo, Japan). Primary antibodies used in this study are shown in **Table S16**.

**Supplementary Figures**

**
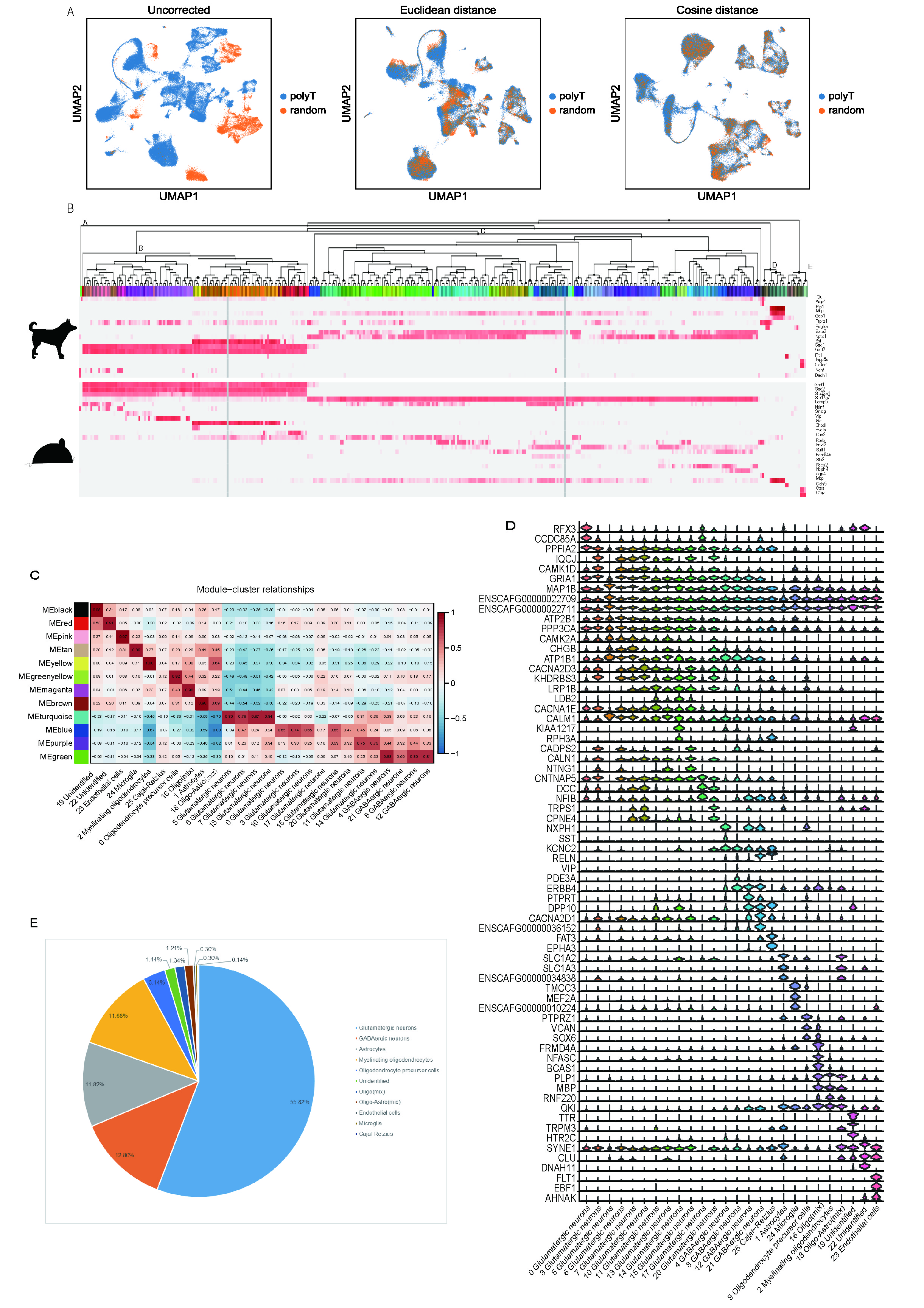
**

**Figure S1.** **Clustering analysis detail information.** (A) The technical effect was corrected by partial PCA and cosine distance. (B) The expression of dog cell types marker genes expressed in mouse brain atlas. Here, 17 genes were taken as dog marker genes in our dog hippocampus data, including astrocytes (*Clu*, *Aqp4*), myelinating oligodendrocytes (*Plp1*, *Mbp*, *Gab1*), oligodendrocyte precursor cells (*Ptprz1*, *Pdgfra*), glutamatergic neurons (*Satb2*, *Nptx1*), GABAergic neurons (*Sst*, *Gad1*, *Gad2*), microglia (*Inpp5d*, *Cx3cr1*), and Cajal-Retzius cells (*Ndnf*, *Dach1*). It also showed 24 mouse different cell types marker genes. Cluster A is Cajal-Retzius cells, cluster B is GABAergic neurons, cluster C is the glutamatergic neurons, cluster D is astrocytes and oligodendrocytes, cluster E is immune and vasculature (© 2015 Allen Institute for Brain Science. Allen Cell Types Database. Available from: <https://celltypes.brain-map.org/rnaseq/mouse_ctx-hip_10x>). (C) Gene modules detected by WGCNA and their enrichment with different cell types. (D) Expression profiles for the DEGs of each cluster. (E) Cell proportion for all the cell types marked with different color.

**
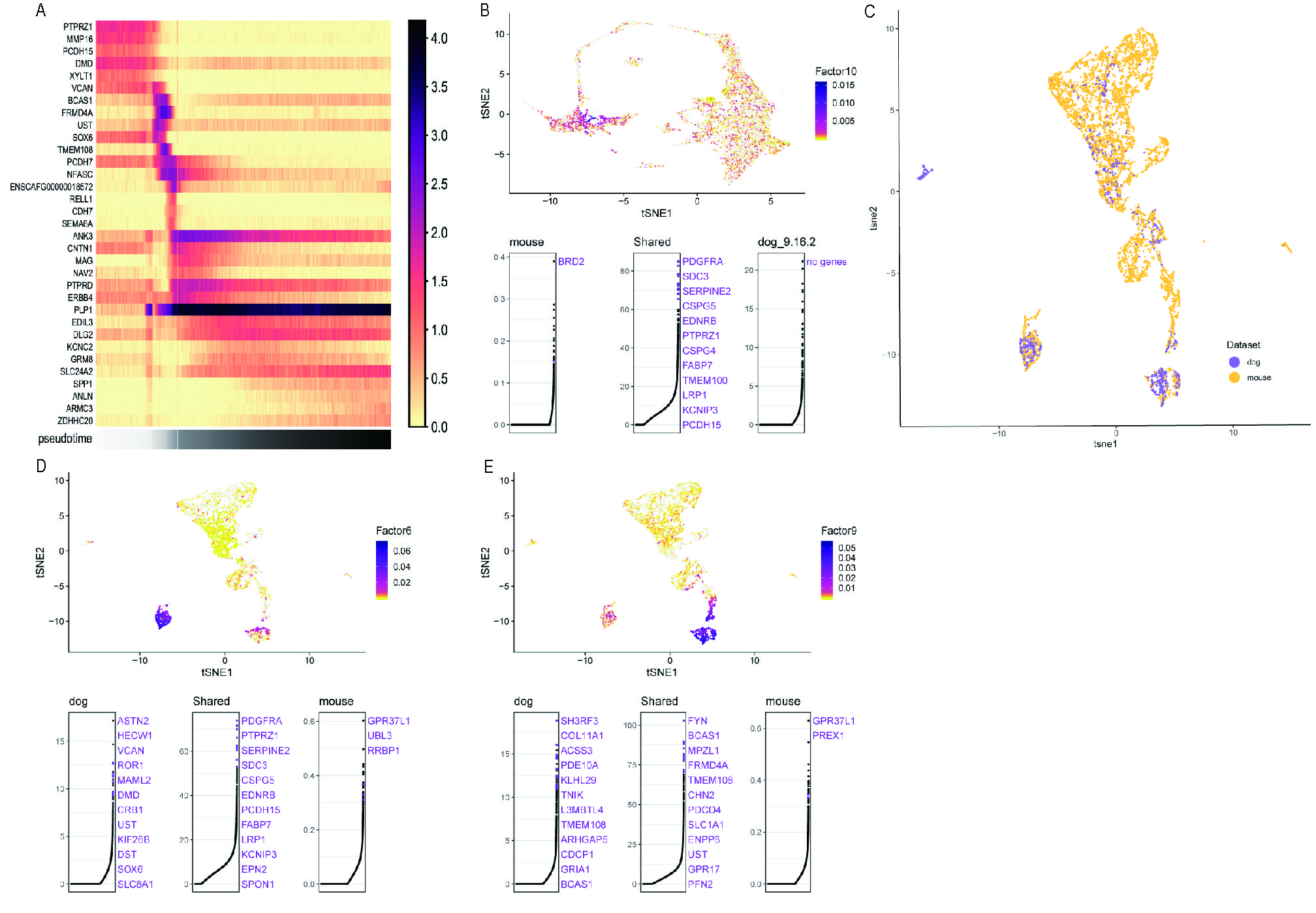
**

**Figure S2. Gene expression patterns of oligodendrocytes and molecular diversity between dog and mice.** (A) Expression heatmap of significantly expressed genes sorted by pseudotime clusters sequence at the bottom. (B) Cell factor loading values (top) and gene loading plots (bottom) show dataset-specific and shared genes for factor 10. In gene loading plots, gene names are sorted in decreasing order of magnitude of their factor loading contribution and correspond to colored points in scatterplots. Plots are organized to show the metagene specific to the dog and mouse and the shared metagenes (*PDGFRA*, *PTPRZ1*) common to two datasets. (C) t-SNE visualization of 6,328 single cells (1,259 from dog and 5,069 from mouse) analyzed by LIGER, color-coded by species. (D–E) UMAP plots showing cell factor loading values (top) and gene loading plots (bottom) for factors corresponding to OPCs and differentiation-committed oligodendrocyte precursors (COPs). (D) Cell factor loading values (top) and gene loading plots (bottom) show dataset-specific and shared genes for factor 6. (E) Cell factor loading values (top) and gene loading plots (bottom) show dataset-specific and shared genes for factor 9. (B-E) Two OPC marker genes were found (*PDGFRA, PTPRZ1*) in both dog and mouse (**Figure S2B**). Since the identity of cluster 16 was unknown, we integrated cluster 16 with mouse cells only for further analysis (**Figure S2C**). As a result, cluster 16 is defined as an intermediate state between mature cells and precursor cells. Specific component factors showed that *PDGFRA* and *FYN* shared in precursor cells (**Figure S2D and E**). However, they are two different precursor cell types’ markers in mouse, which are OPCs and differentiation-committed oligodendrocyte precursors (COPs), and COPs lacked *PDGFRA* and *CSPG4* [24].

**
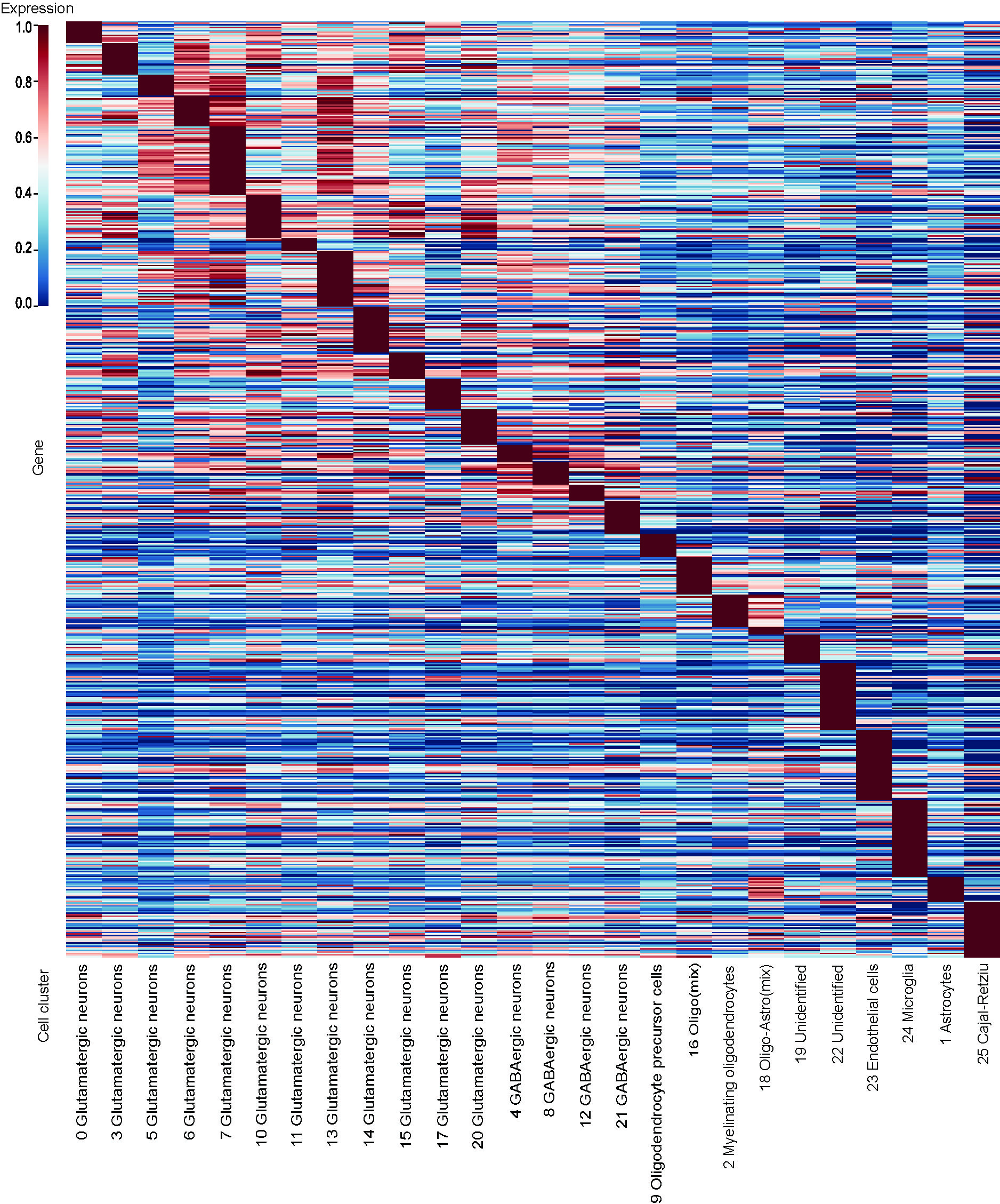
**

**Figure S3. The mean expression of 630 putative PSGs in different cell types.** The values of each gene (row) were its original ones divided by its maximum. This figure related to **Table S6.**


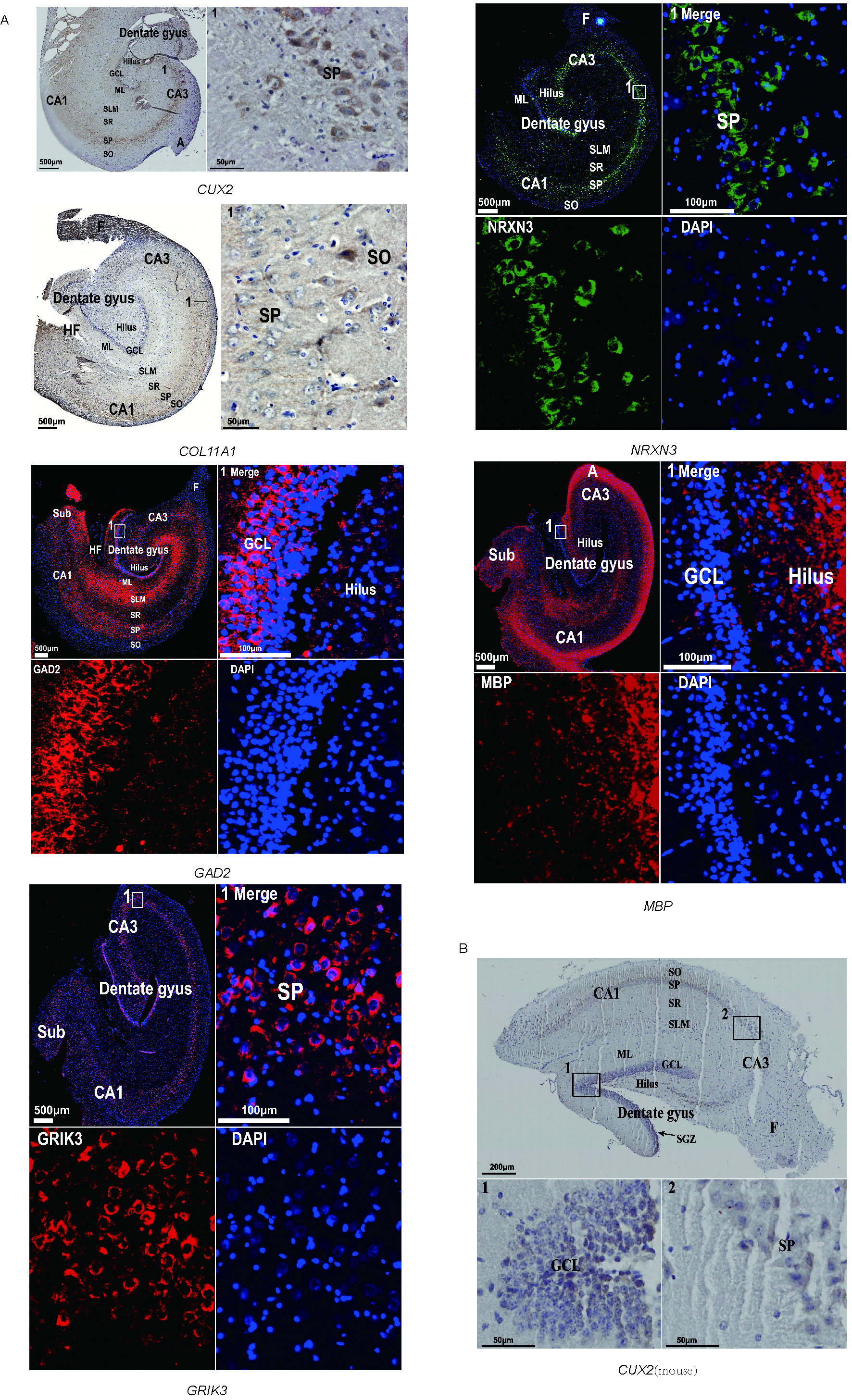


**Figure S4. High-definition immunohistochemical staining** (this figure is related to **Figure 4B)**. (A) Immunohistochemical staining for *CUX2*, *COL11A1*, *GAD2*, *GRIK3*, *NRXN3*, *MBP*.

(B) Visualization of *CUX2* expression in mouse hippocampus by immunohistochemical staining. The expression of *CUX2* in the mouse hippocampus is the strongest in the subgranular zone (progenitor cell-enriched in this area) and hilus of DG cells, it is lowly expressed in CA3 and CA1 granular cells.

**Reference**

1. Rosenberg AB, Roco CM, Muscat RA, et al.; Single-cell profiling of the developing mouse brain and spinal cord with split-pool barcoding. *Science* 2018;**360**(6385):176-+. doi: 10.1126/science.aam8999.

2. Wolf FA, Angerer P, Theis FJ; SCANPY: large-scale single-cell gene expression data analysis. *Genome Biology* 2018;**19**. doi: 10.1186/s13059-017-1382-0.

3. Mcinnes L, Healy J; UMAP: Uniform Manifold Approximation and Projection for Dimension Reduction. *The Journal of Open Source Software* 2018;**3**(29):861.

4. Traag VA, Waltman L, van Eck NJ; From Louvain to Leiden: guaranteeing well-connected communities. *Sci Rep* 2019;**9**(1):5233. doi: 10.1038/s41598-019-41695-z.

5. Wilcoxin F; Probability tables for individual comparisons by ranking methods. *Biometrics* 1947;**3**(3):119-22.

6. Finak G, McDavid A, Yajima M, et al.; MAST: a flexible statistical framework for assessing transcriptional changes and characterizing heterogeneity in single-cell RNA sequencing data. *Genome Biology* 2015;**16**. doi: 10.1186/s13059-015-0844-5.

7. Yu GC, Wang LG, Han YY, et al.; clusterProfiler: an R Package for Comparing Biological Themes Among Gene Clusters. *Omics-a Journal of Integrative Biology* 2012;**16**(5):284-287. doi: 10.1089/omi.2011.0118.

8. Carlson M; org.Cf.eg.db: Genome wide annotation for Canine. R package version 3.10.0. . 2019.

9. Langfelder P, Horvath S; WGCNA: an R package for weighted correlation network analysis. *Bmc Bioinformatics* 2008;**9**. doi: 10.1186/1471-2105-9-559.

10. Zhong SJ, Ding WY, Sun L, et al.; Decoding the development of the human hippocampus. *Nature* 2020;**577**(7791):531-536. doi: 10.1038/s41586-019-1917-5.

11. Cunningham F, Allen JE, Allen J, et al.; Ensembl 2022. *Nucleic Acids Res* 2022;**50**(D1):D988-D995. doi: 10.1093/nar/gkab1049.

12. Liu X, Shen Q, Zhang S; Cross-species cell-type assignment of single-cell RNA-seq by a heterogeneous graph neural network. *bioRxiv* 2021.

13. Welch JD, Kozareva V, Ferreira A, et al.; Single-Cell Multi-omic Integration Compares and Contrasts Features of Brain Cell Identity. *Cell* 2019;**177**(7):1873-1887. doi: 10.1016/j.cell.2019.05.006.

14. Axelsson E, Ratnakumar A, Arendt ML, et al.; The genomic signature of dog domestication reveals adaptation to a starch-rich diet. *Nature* 2013;**495**(7441):360-364. doi: 10.1038/nature11837.

15. Freedman AH, Schweizer RM, Ortega-Del Vecchyo D, et al.; Demographically-Based Evaluation of Genomic Regions under Selection in Domestic Dogs. *Plos Genetics* 2016;**12**(3). doi: 10.1371/journal.pgen.1005851.

16. Pendleton AL, Shen FC, Taravella AM, et al.; Comparison of village dog and wolf genomes highlights the role of the neural crest in dog domestication. *Bmc Biology* 2018;**16**. doi: 10.1186/s12915-018-0535-2.

17. vonHoldt BM, Pollinger JP, Lohmueller KE, et al.; Genome-wide SNP and haplotype analyses reveal a rich history underlying dog domestication. *Nature* 2010;**464**(7290):898-U109. doi: 10.1038/nature08837.

18. Wang GD, Zhai WW, Yang HC, et al.; Out of southern East Asia: the natural history of domestic dogs across the world. *Cell Research* 2016;**26**(1):21-33. doi: 10.1038/cr.2015.147.

19. Martinez O, Reyes-Valdes MH; Defining diversity, specialization, and gene specificity in transcriptomes through information theory. *Proceedings of the National Academy of Sciences of the United States of America* 2008;**105**(28):9709-9714. doi: 10.1073/pnas.0803479105.

20. Zhang JTD, Hatje K, Sturm G, et al.; Detect tissue heterogeneity in gene expression data with BioQC. *Bmc Genomics* 2017;**18**. doi: 10.1186/s12864-017-3661-2.

21. Huynh-Thu VA, Irrthum A, Wehenkel L, et al.; Inferring Regulatory Networks from Expression Data Using Tree-Based Methods. *Plos One* 2010;**5**(9). doi: 10.1371/journal.pone.0012776.

22. Hu H, Miao YR, Jia LH, et al.; AnimalTFDB 3.0: a comprehensive resource for annotation and prediction of animal transcription factors. *Nucleic Acids Research* 2019;**47**(D1):D33-D38. doi: 10.1093/nar/gky822.

23. Raudvere U, Kolberg L, Kuzmin I, et al.; g:Profiler: a web server for functional enrichment analysis and conversions of gene lists (2019 update). *Nucleic Acids Research* 2019;**47**(W1):W191-W198. doi: 10.1093/nar/gkz369.

24. Marques S, Zeisel A, Codeluppi S, et al.; Oligodendrocyte heterogeneity in the mouse juvenile and adult central nervous system. *Science* 2016;**352**(6291):1326-1329. doi: 10.1126/science.aaf6463.
